# Effects of GIP on regional blood flow during normoglycemia and hyperglycemia in anesthetized rats

**DOI:** 10.14814/phy2.13685

**Published:** 2018-04-19

**Authors:** Xiang Gao, Andreas Lindqvist, Monica Sandberg, Leif Groop, Nils Wierup, Leif Jansson

**Affiliations:** ^1^ Department of Medical Cell Biology Uppsala University Uppsala Sweden; ^2^ Department of Clinical Sciences Lund University Diabetes Centre Lund University Malmö Sweden

**Keywords:** Glucose‐dependent insulinotropic peptide, incretin hormones, Islet blood flow, pancreatic islets, splanchnic blood flow

## Abstract

The incretin hormone glucose‐dependent insulinotropic polypeptide (GIP) potentiates glucose‐stimulated insulin secretion, and affects *β*‐cell turnover. This study aimed at evaluating if some of the beneficial effects of GIP on glucose homeostasis can be explained by modulation of islet blood flow. Anesthetized Sprague–Dawley rats were infused intravenously with different doses of GIP (10, 20, or 60 ng/kg*min) for 30 min. Subsequent organ blood flow measurements were performed with microspheres. In separate animals, islets were perfused ex vivo with GIP (10^−6^–10^−12^ mol/L) during normo‐ and hyperglycemia and arteriolar responsiveness was recorded. The highest dose of GIP potentiated insulin secretion during hyperglycemia, but had no effect in normoglycemic rats. The highest GIP concentration decreased blood perfusion of whole pancreas, pancreatic islets, duodenum, colon, liver and kidneys. The decrease in blood flow was unaffected by ganglion blockade or adenosine receptor inhibition. In contrast to this, in single perfused islets GIP induced a dose‐dependent arteriolar dilation. Thus, high doses of GIP exert a direct dilatory effect on islet arterioles in isolated islets, but induce a generalized vasoconstriction in splanchnic organs, including the whole pancreas and islets, in vivo. The latter effect is unlikely to be mediated by adenosine, the autonomic nervous system, or endothelial mediators.

## Introduction

Incretins are gut‐derived hormones which potentiate glucose‐stimulated insulin release after a meal (Drucker 2013). The major incretins are glucagon‐like peptide‐1 (GLP‐1), produced by L‐cells, and glucose‐dependent insulinotropic polypeptide (GIP), produced by K‐cells. Their effects follow hormone plasma concentrations and are fairly short‐acting, since they are rapidly degraded by the ubiquitous exopeptidase dipeptidyl peptidase‐4 (DPP‐4) and they are also cleared by the kidneys (Holst and Gromada 2004). Both incretins act via specific receptors, which are present on *β*‐cells, and numerous other cell types in the body. The receptors induce cAMP formation, thereby potentiating only glucose‐stimulated, and not basal insulin secretion (Holst 2004; Holst and Gromada 2004). GLP‐1 inhibits glucagon secretion, whereas GIP on the other hand stimulates glucagon secretion (Lyssenko et al. 2011). Also, both incretins stimulate *β*‐cell proliferation and inhibit *β*‐cell apoptosis. As the name suggests, GIP acts as an incretin hormone, particularly at elevated levels of glucose in the circulation and in the presence of cholinergic agonists (Muller et al. 1986; Fehmann et al. 1995). GIP secretion is mainly stimulated by nutrient ingestion (Fehmann et al. 1995; Yip and Wolfe 2000). GIP receptors (GIPR) are widely distributed and found in, for example, pancreatic islet cells (Lyssenko et al. 2011) and endothelium (Usdin et al. 1993). Single nucleotide polymorphisms (SNPs) in the *gipr* gene have been reported to be associated with impaired glucose‐ and GIP‐stimulated insulin secretion (Lyssenko et al. 2011).

Since both GLP‐1‐receptor agonists and DPP‐4 inhibitors are clinically used, great attention has been given to elucidate GLP‐1‐mediated modes of action. GIP, on the other hand, has received less attention even though its receptor is well characterized. Also, it has been suggested that GIP could have a place as an adjunct treatment of type 2 diabetes (T2D) if added to insulin but concern has been raised because of its ability to increase fat deposition in adipose tissue and thereby body weight (Ussher and Drucker 2012; Campbell and Drucker 2013; Drucker 2013). Incretins, particularly GLP‐1, have also been ascribed a key role in the rapid improvement of glucose homeostasis after Roux‐en‐Y gastric bypass (RYGB) surgery in T2D and nondiabetic obese patients. However, less is known about the role of GIP with reports of mainly increased (Lindqvist et al. 2013, 2017; Berggren et al. 2017; Honka et al. 2017) but in some cases unchanged (Svane et al. 2016) GIP levels.

Since the incretin receptors are present also on vascular cells, incretins may have a direct effect on circulation. We have previously shown that pancreatic islets regulate their blood flow separately from that of the exocrine parenchyma of the pancreas. We have also observed that elevated glucose, as seen in impaired glucose tolerance and overt T2D in rodents, induces an increase in islet blood flow, which may have adverse affects on the islets per se (Jansson 1994; Jansson and Carlsson 2002). It is thus possible that effects on pancreatic islet vasculature may also affect the endocrine function. In support of this, we have observed that most drugs used to treat diabetes in experimental animal models increase islet blood flow in control animals, but normalize islet blood flow in T2D animals (Jansson 1994; Carlsson et al. 2003). It may be that incretins exert additional beneficial effects on pancreatic islet function through actions on islet blood flow.

In support of this hypothesis, we have observed that GLP‐1 and its analog exenatide affects pancreatic islet blood flow and reduces the augmented islet blood flow seen in animal models of T2D (Svensson et al. 2007; Wu et al. 2012b). In a previous study on effects of GIP in lean rats, we observed that a bolus dose of GIP dose‐dependently enhanced islet blood flow during acute hyperglycemia in normal rats (Svensson et al. 1997; Ruttimann et al. 2009). Thus, there seems to be inconsistent findings on the effects of incretin hormones on islet blood flow. This study was designed to address the effect of different doses of systemic and portal infusion of GIP on pancreatic and splanchnic blood flow. We also applied pharmacological manipulations to elucidate the involvement of the nervous system.

## Materials and Methods

### Animals

Male Sprague–Dawley rats (Scanbur, Sollentuna, Sweden) weighing 300–350 g with free access to standard rat chow and tap water were used in the experiments. All experiments were approved by the local animal ethics committee at Uppsala University.

### Chemicals

Chemicals were purchased from Sigma‐Aldrich (St. Louis, MO) unless otherwise stated.

### Infusion of GIP

Rats were anesthetized with an intraperitoneal injection of thiobutabarbital sodium (120 mg/kg body weight; Inactin^®^), after which the animals were placed on a heated operating table to maintain body temperature at 37.5°C. A polyethylene catheter was inserted into the ascending aorta, via the right carotid artery, and separate catheters were placed into the left femoral artery and vein, respectively. The femoral artery catheter was connected to a pressure transducer (Physiologic Pressure Transducer; AD Instruments, Oxford, UK), whereas the venous catheter was used for infusions with Ringer solution containing 0.1% (w/v) bovine serum albumin (4 mL/kg body weight*h) with or without addition of GIP as outlined below. After an equilibrium period when all animals were infused with Ringer solution for 20 min, human GIP was added to the Ringer solution in some animals (10, 20 or 60 ng/kg*min) for an additional 30 min. At 27 min after beginning, the infusion a bolus dose of 1 mL of saline or 30% (w/v) d‐glucose was injected intravenously. Three min later blood flow measurements were performed using the microsphere technique (see below).

The same protocol as described above was followed in other groups of rats, but only normoglycemic rats were used. Twenty minutes into the infusion of saline or GIP (60 ng/kg*min) a single intravenous dose of theophylline (a nonspecific adenosine antagonist; 6 mg/kg) or hexamethonium (a ganglion blocker; 10 mg/kg) was given. Blood flow measurements were then performed 30 min after initiating the saline‐ or GIP infusion.

### Blood flow measurements with microspheres

This has been detailed elsewhere (Carlsson et al. 2002a; Jansson et al. 2016). A total of 3 × 10^5^ black nonradioactive microspheres (EZ‐Trac™; Triton Microspheres), with a diameter of 10 *μ*m were injected via the catheter with its tip in the ascending aorta during 10 s whole simultaneously collecting an arterial reference sample (Carlsson et al. 2002a). Arterial blood was collected from the carotid catheter for determination of hematocrit, glucose concentrations with test reagent strips (MediSense AB, Sollentuna, Sweden) and serum insulin concentrations with an ELISA (Mercodia AB, Uppsala, Sweden). GIP was measured in plasma collected into chilled EDTA‐tubes (500 KIU/mL aprotinin) using an ELISA from Millipore (Billerica, MA). This assay measures total GIP (GIP_1‐42_ and GIP_3‐42_) and was performed according to the instructions provided by the manufacturer. In our GIP‐infused control rats, the GIP concentrations increased in accordance to the administered dose of GIP, suggesting that also human GIP was detected.

The rats were then killed and the pancreas and adrenal glands were removed in toto, blotted and weighed. Samples (approximately 100 mg) from the mid‐regions of the duodenum, descending the colon and left kidney were also removed, blotted, and weighed. The number of microspheres in the samples referred to above, including the pancreatic islets, was counted in a microscope equipped with both bright and dark field illumination after treating the organs with freezing–thawing (Jansson and Hellerström 1981). The number of microspheres in the arterial reference sample was separately determined. The organ blood flow values were calculated based in microsphere content as previously described (Jansson and Hellerström 1983). A difference <10% in the adrenal blood flow values was taken to indicate sufficient mixing of microspheres with blood, and this occurred in all animals in the present study (data not shown).

### Blood flow measurements after intraportal infusions

The animals were prepared for blood flow measurements as described above. The upper part of the abdominal cavity was opened via a midline incision and the portal vein immediately before its entry to the liver hilus was visualized by gently moving the intestines upwards and to the left (Carlsson et al. 2000). A catheter was then placed into the portal vein and used to infuse (1 mL/h) saline alone or with GIP (60 ng/min per kg) during 30 min. After 27 min, 1 mL of saline or 30% (w/v) d‐glucose was injected intravenously. Three min later the blood flow measurements were performed.

### Isolation and preparation of islets for single islet arteriolar responsiveness

Rats were killed by an overdose of pentobarbital, and the pancreas was quickly removed and placed in cold (4°C) bovine serum albumin‐enriched (1%) Dulbecco's Minimal Enriched medium (DMEM). Islets were dissected with their arterioles intact (Lai et al. 2007a) using a modification of a previously described technique for renal glomeruli (Patzak et al. 2001, 2004). The time for dissection was limited to 60 min and the obtained islets had diameters of approximately 200 *μ*m. For further details, see previous publications (Lai et al. 2007a,b). The experimental setup allowed for measurement of the diameter of the afferent islet arterioles continuously and the recording of changes at a resolution of < 0.2 *μ*m.

Each experiment began with a 15‐min equilibrium period with buffer containing 5.5 mmol/L glucose in both bath and perfusion solutions. Thereafter, 16.7 mmol/L of glucose was added to both bath and perfusion medium together with increasing doses of GIP (from 10^−12^ to 10^−6^ mol/L) in 2‐min intervals. Thereafter, a new 15‐min equilibrium period with 5.5 mmol/L of glucose was run followed by administration of increasing doses of GIP at the same concentrations and intervals as given above but with 5.5 mmol/L glucose. Each perfusion was terminated by administration of KCl (100 mmol/L) to ascertain that the arterioles were able to contract.

### Statistical analyses

All values are given as mean ± SEM. Probabilities (*P*) of chance differences were calculated with a Student's unpaired *t*‐test or one‐way repeated measurement ANOVA with Tukey's correction (SigmaStat™; SSPD, Erfart, Germany). A value of *P* < 0.05 was considered to be statistically significant.

## Results

**Figure 1:**
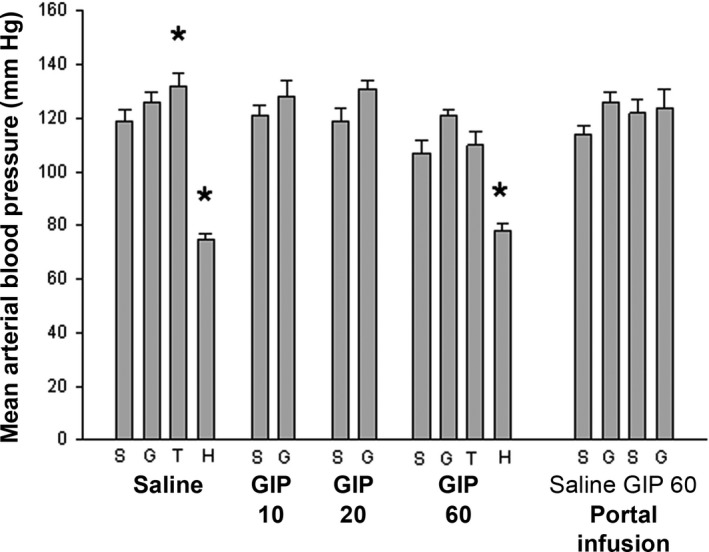
Mean arterial blood pressure in anesthetized Sprague–Dawley rats. The rats were infused with Ringer solution (Saline) with or without GIP (10, 20 or 60 ng/kg*min) for 30 min. After 20 min of infusion, some rats were given a bolus dose of theophylline (T; 6 mg/kg in Ringer) or hexamethonium (H; 10 mg/kg). Other rats were given a bolus dose of 1 mL Ringer (S) or 30% d‐glucose (G) after 27 min of infusion. Blood flow measurements were made at 30 min with a microsphere technique. In some rats, infusions (1 mL/h for 30 min) were made directly into the portal vein (Portal infusion) of Ringer alone or with added GIP (60 ng/kg*min). These animals were also a given bolus dose of 1 mL Ringer (S) or 30% d‐glucose (G) after 27 min of infusion. Values are mean ± SEM for 7–8 experiments. *Denotes *P* < 0.01 when compared to the corresponding Ringer‐injected rat. GIP: glucose‐dependent insulinotropic polypeptide.

The mean body weight of the rats was 343 ± 5 g (*n* = 126). Hematocrit values were similar in all saline‐infused groups at 44–46% and decreased 2–4% after glucose administration in all groups (data not shown). The mean arterial blood pressure varied between 105 and 120 mmHg in all groups except animals given hexamethonium where the pressure decreased to 75–80 mmHg (Fig. 1).

### Glucose and hormone concentrations

**Figure 2:**
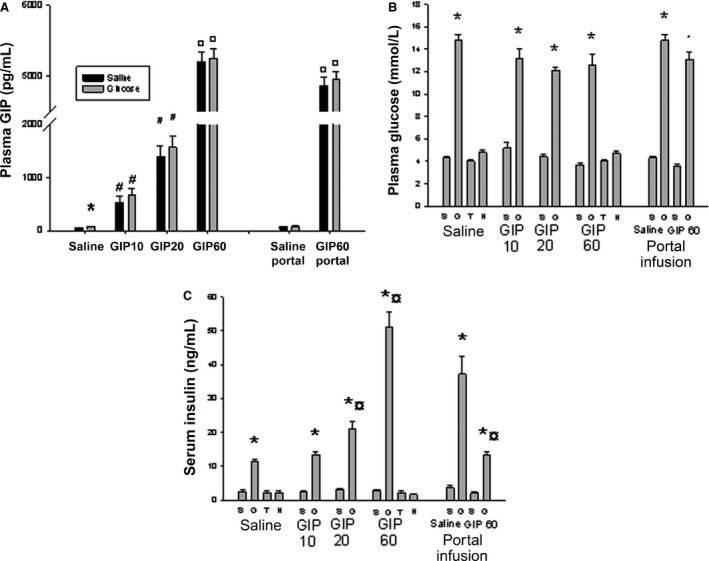
Plasma concentrations of GIP were found to be dose‐dependently increased after infusion of GIP in both normo‐ and hyperglycemic rats (A). *P < 0.05; #*P* < 0.01; ¤ *P* < 0.0001 compared to normoglycemic rats receiving saline infusions. Plasma glucose (B) and plasma insulin concentrations (C) in anesthetized Sprague–Dawley rats. The rats were infused with Ringer solution (Saline) with or without GIP (10, 20 or 60 ng/kg*min) for 30 min. After 20 min of infusion, some rats were given a bolus dose of theophylline (T; 6 mg/kg (BW) in Ringer) or hexamethonium (H; 10 mg/kg BW). Other rats were given a bolus dose of 1 mL Ringer (S) or 30% d‐glucose (G) after 27 min of infusion. Blood flow measurements were made at 30 min with a microsphere technique. In some rats infusions (1 mL/h for 30 min) were made directly into the portal vein (Portal infusion) of Ringer alone or with added GIP (60 ng/kg*min). These animals were also a given bolus dose of 1 mL Ringer (S) or 30% d‐glucose (G) after 27 min of infusion. Values are mean ± SEM for 7–8 experiments. *Denotes *P* < 0.01 when compared to the corresponding Ringer‐injected rat. ^**¤**^Denotes *P* < 0.05 when compared to the corresponding Ringer‐infused group. GIP: glucose‐dependent insulinotropic polypeptide.

GIP infusions induced the expected increases in plasma GIP concentrations in all groups throughout the experiments with no difference between normo‐ and hyperglycemic animals (Fig. 2A). Basal GIP concentrations were in the picomolar range (Fig. 2A).

Glucose administration increased plasma glucose concentrations in all groups (Fig. 2B). GIP infusion, systemic or intraportal, did not further influence plasma glucose neither in normo‐ nor in hyperglycemic rats (Fig. 2B). Also, neither theophylline nor hexamethonium affected plasma glucose concentrations (Fig. 2B).

No significant effects of systemic or intraportal GIP administration or theophylline and hexamethonium were observed on serum insulin concentrations in the normoglycemic animals (Fig. 2C). As expected glucose administration increased insulin concentrations; this increase was further potentiated by the two highest doses of GIP given into the femoral vein (Fig. 2C). After intraportal infusion of GIP, acute hyperglycemia increased insulin concentrations. This response was lower than in normoglycemic control rats (Fig. 2C). It should be noted that serum insulin concentrations were markedly higher when the same amount of glucose was infused intraportally, compared to after systemic infusion (*P* < 0.01).

### Pancreatic and islet blood flow

**Figure 3:**
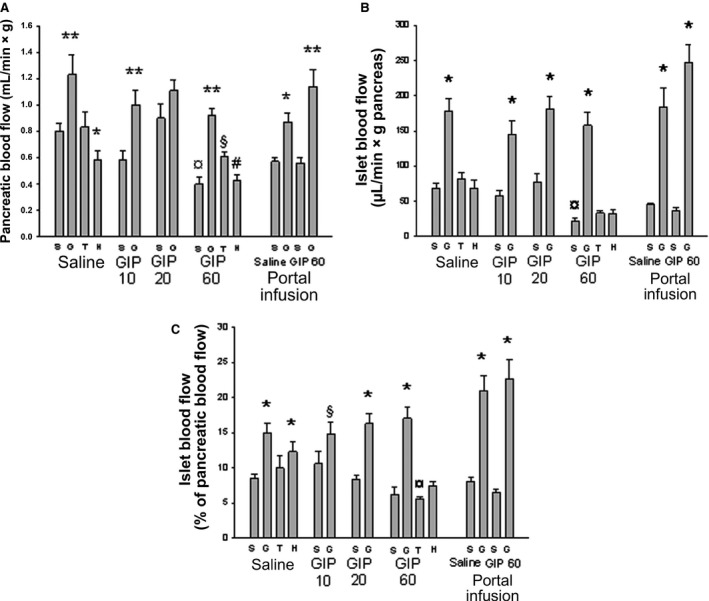
Total pancreatic blood flow (A), islet blood flow (B), and fractional islet blood flow (C; i.e., the fraction of total pancreatic blood flow diverted through the islets) in anesthetized Sprague–Dawley rats. The rats were infused with Ringer solution (Saline) with or without GIP (10, 20 or 60 ng/kg*min) for 30 min. After 20 min of infusion, some rats were given a bolus dose of theophylline (T; 6 mg/kg BW) or hexamethonium (H; 10 mg/kg BW). Other rats were given a bolus dose of 1 mL Ringer (S) or 30% d‐glucose (G) after 27 min of infusion. Blood flow measurements were made at 30 min with a microsphere technique. In some rats infusions (1 mL/h for 30 min) were made directly into the portal vein (Portal infusion) of Ringer alone or with added GIP (60 ng/kg*min). These animals were also a given bolus dose of 1 mL Ringer (S) or 30% d‐glucose (G) after 27 min of infusion. Values are means ± SEM for 7–8 experiments. *Denotes *P* < 0.01 when compared to the corresponding Ringer‐injected rat. ^**¤**^Denotes *P* < 0.05 when compared to the corresponding Ringer‐infused group. #Denotes *P* < 0.05 when compared to the other glucose‐injected groups. GIP: glucose‐dependent insulinotropic polypeptide.

In saline‐infused control rats, acute hyperglycemia increased total pancreatic (Fig. 3A), islet (Fig. 3B) and fractional islet blood flow (Fig. 3C). Theophylline had no effects on flow values in normoglycemic rats. Since hexamethonium decreased total pancreatic blood flow while leaving islet blood flow unchanged, this resulted in increased fractional islet blood flow. In contrast, infusion of GIP (60 ng/kg*min) decreased both total pancreatic (Fig. 3A) and islet blood flow (Fig. 3B) to the same extent, thereby maintaining fractional islet blood flow unchanged (Fig. 3C). Neither theophylline nor hexamethonium prevented the effects of the highest dose of GIP on pancreatic blood flow. The lower doses of GIP had no effect on blood flow in normo‐ or hyperglycemic rats, compared to saline‐infused control rats.

Intraportal infusion of GIP (60 ng/min*kg) had no effects on blood flow in either normo‐ or hyperglycemic rats. Also, there was no difference compared with systemic GIP infusion of control rats.

### Blood flow in duodenum and colon

**Figure 4:**
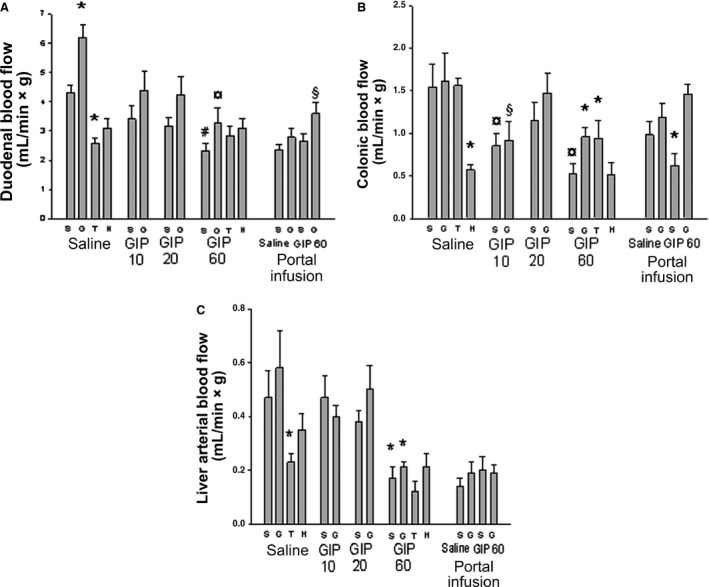
Duodenal blood flow (A), colonic blood flow (B), and liver arterial blood flow (C) in anesthetized Sprague–Dawley rats. The rats were infused with Ringer solution (Saline) with or without GIP (10, 20 or 60 ng/kg*min) for 30 min. After 20 min of infusion some rats were given a bolus dose of theophylline (T; 6 mg/kg BW) in Ringer) or hexamethonium (H; 10 mg/kg BW). Other rats were given a bolus dose of 1 mL Ringer (S) or 30% d‐glucose (G) after 27 min of infusion. Blood flow measurements were made at 30 min with a microsphere technique. In some rats infusions (1 mL/h for 30 min) were made directly into the portal vein (Portal infusion) of Ringer alone or with added GIP (60 ng/kg*min). These animals were also a given bolus dose of 1 mL Ringer (S) or 30% d‐glucose (G) after 27 min of infusion. Values are mean ± SEM for 7–8 experiments. *Denotes *P* < 0.01 when compared to the corresponding Ringer‐injected rat. ^**¤**^Denotes *P* < 0.05 when compared to the corresponding Ringer‐infused group. #Denotes *P* < 0.05 when compared to the other glucose‐injected groups. GIP: glucose‐dependent insulinotropic polypeptide.

To dissect whether the effect of GIP on pancreatic blood flow was more general or restricted to pancreas, we also compared the effect on blood flow in duodenum (receiving its blood supply from the superior mesenteric artery in the same way as the pancreatic artery) and colon (which is supplied by the inferior mesenteric artery). Hyperglycemia per se increased duodenal blood flow in saline‐infused rats, whereas theophylline decreased duodenal flow (Fig. 4A). Hexamethonium had no effect on basal duodenal blood flow; a trend (*P* = 0.07) towards a decrease was observed. The highest dose of GIP (60 ng/kg*min) decreased duodenal blood flow in both normo‐ and hyperglycemic rats compared to corresponding saline‐infused controls. Both duodenal and colonic blood flow decreased when the abdomen was opened for intraportal infusions (*P* < 0.02 compared to saline‐infused controls). When the highest dose of GIP (60 ng/kg per minute) was infused intraportally into hyperglycemic rats, an increase in duodenal blood flow was observed; this was not seen in normoglycemic rats.

In saline‐infused controls hexamethonium caused a pronounced decrease in colonic blood flow, whereas hyperglycemia and theophylline administration had no effects (Fig. 4B). GIP at doses of 10 or 60 ng/min*kg caused a decrease in colonic blood flow in both normo‐ and hyperglycemic rats. GIP effects on colonic blood flow were unaffected by theophylline administration. Intraportal GIP infusion decreased colonic blood flow in normoglycemic but not in hyperglycemic rats.

### Hepatic artery flow

Hepatic arterial blood flow was decreased by theophylline in saline‐infused control rats, whereas hyperglycemia and hexamethonium had no effect (Fig. 4C). The highest dose of GIP (60 ng/kg*min) decreased hepatic arterial blood flow in both normo‐ and hyperglycemic rats. This effect was further potentiated by theophylline, but not by hexamethonium. Intraportal infusions in general decreased hepatic arterial blood flow compared to control rats infused systemically with saline (*P* < 0.01). No further effect of intraportally infused GIP was observed on hepatic arterial blood flow.

### Renal and adrenal blood flow

**Figure 5:**
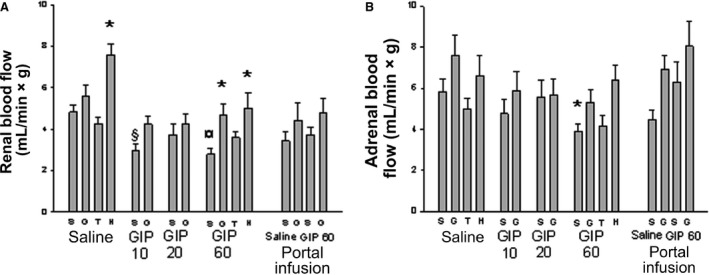
Renal blood flow (A) and blood flow (B) in anesthetized Sprague–Dawley rats. The rats were infused with Ringer solution (Saline) with or without GIP (10, 20, or 60 ng/kg*min) for 30 min. After 20 min of infusion some rats were given a bolus dose of theophylline (T; 6 mg/kg BW) in Ringer) or hexamethonium (H; 10 mg/kg BW). Other rats were given a bolus dose of 1 mL Ringer (S) or 30% d‐glucose (G) after 27 min of infusion. Blood flow measurements were made at 30 min with a microsphere technique. In some rats, infusions (1 mL/h for 30 min) were made directly into the portal vein (Portal infusion) of Ringer alone or with added GIP (60 ng/kg*min). These animals were also a given bolus dose of 1 mL Ringer (S) or 30% d‐glucose (G) after 27 min of infusion. Values are mean ± SEM for 7–8 experiments. *Denotes *P* < 0.01 when compared to the corresponding Ringer‐injected animal. ^**¤**^Denotes *P* < 0.05 when compared to the corresponding Ringer‐infused group. GIP: glucose‐dependent insulinotropic polypeptide.

In saline‐infused control rats hexamethonium caused a pronounced increase in renal blood flow, whereas hyperglycemia and theophylline administration had no effect (Fig. 5A). The highest dose of GIP (60 ng/kg*min) decreased renal blood flow in normoglycemic rats as did the GIP dose 10 ng/min*kg. The dose of 20 ng/kg*min had no effect. The decrease in renal blood flow seen with the highest dose of GIP was prevented by glucose and hexamethonium administration.

Only the highest dose of GIP (60 ng/kg*min) decreased adrenal blood flow in normoglycemic rats (Fig. 5B).

### Islet arteriolar vascular reactivity

**Figure 6:**
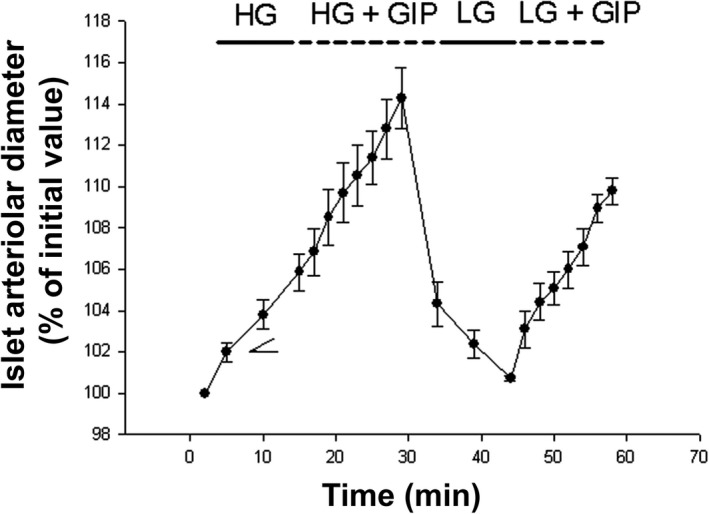
Changes in the diameter of islet arterioles isolated from normoglycemic Sprague–Dawley rats after administration of buffer (DMEM + 3% bovine serum albumin and 2.8 mmol/L [LG] or 16.7 mmol/L [HG] at a pressure of 70 mmHg. The medium contained different concentrations of GIP. The control value initiating the experiments represents the value for buffer without any added glucose. Values are given as per cent of the diameter before administration of the test substances (original diameter 32 ± 3 *μ*m; *n* = 11). GIP: glucose‐dependent insulinotropic polypeptide.

In arterioles of isolated islets, high (16.7 mmol/L) but not low (2.8 mmol/L) glucose concentrations caused a 6–7% dilation of arteriolar vascular smooth muscle, (Fig. 6). When GIP at different concentrations (10^−12^–10^−6^ mol/L) was added to both the low‐ and high‐glucose‐containing medium, a dose‐dependent increase in dilation of up to 11–12% was seen. All islet arterioles reacted with a pronounced vasoconstriction to KCl at the end of the experiments (data not shown), which thereby confirmed their viability.

## Discussion

The main new findings in this study are that high, supraphysiological GIP concentrations decreased the blood flow in whole pancreas, pancreatic islets, duodenum, colon, liver, and kidneys thereby indicating a similar effect on blood flow in organs supplied by different arteries. This flow decrease was unaffected by ganglion blockade with hexamethonium (except in the kidney) and adenosine receptor inhibition with theophylline and thereby unlikely to involve endothelial mediators. In contrast to these in vivo findings single perfused islet arterioles showed a dose‐dependent dilation in response to GIP suggesting that GIP exerts a direct dilatory effect on arteriolar smooth muscle cells in isolated islets, but vasoconstriction in vivo in intact animals. The consequences for insulin secretion and glucose metabolism of this we do not yet know, but it may be that it directly affects glucose homeostasis. It has indeed been previously suggested that a prevented vasodilation may be protective for islet function during conditions with impaired glucose tolerance (Jansson et al. 2016).As expected, GIP had no effects on plasma glucose or serum insulin concentrations in normoglycemic animals (Holst and Gromada 2004). Neither did the ganglion blocker hexamethonium nor theophylline, an adenosine receptor antagonist in the used concentration (Carlsson et al. 2002b), affect glucose or insulin. In contrast, during hyperglycemia the highest GIP‐dose potentiated glucose‐induced insulin secretion. Our findings on GIP effects on glucose homeostasis were thus in line with previous studies and confirm the physiological effects of the used doses (Svensson et al. 2000, 2005).

Although it is well established that incretins (mainly GLP‐1) and DPP‐4 inhibitors, exert multiple cardioprotective actions (Ussher and Drucker 2012), we did not observe any effects on the mean arterial blood pressure by GIP, suggesting no generalized vascular effect. However, we noted several changes in local blood flow that may be of interest for the actions and possible benefits of GIP.

Infusion of GIP at 60 ng/kg*min decreased total pancreatic and islet blood flow to the same extent in normoglycemic rats, whereas lower doses (10 and 20 ng/kg*min) had no effect. Acute hyperglycemia (Carlsson et al. 2002b) increased both islet and total pancreatic blood flow as expected. This increase could be prevented, either completely by the ganglion blocker hexamethonium or partially by adenosine receptor inhibition. These findings confirm our previous observation that islet blood flow is increased during hyperglycemia by both nervous and metabolic signals (Carlsson et al. 2002b). Interestingly, the blood flow responses to hyperglycemia were largely unaffected by all doses of GIP. These findings should be considered in the light of our previous observations (Svensson et al. 1997) where a high bolus dose (15 *μ*g/kg) of GIP 10 min before blood flow measurements decreased total pancreatic and duodenal blood flow, whereas islet blood flow remained unaffected. In contrast to the present study an enhanced islet blood flow during hyperglycemia was observed (Jansson and Hellerström 1981). These differences might be ascribed to the differences in administration of GIP, viz. bolus versus infusion; the latter yielding higher and more constant GIP concentrations. After a bolus, one should expect a time‐dependent degradation of GIP. Therefore, the present results most likely reflect a more continuous situation representing GIP secretion after a meal. This is also supported by previous and similar findings when administering GLP‐1 (Svensson et al. 2007). This hormone had no effect on baseline blood flow, but prevented glucose‐induced increase in islet blood flow. The GLP‐1 analogue liraglutide improved glucose tolerance in GK rats, a T2D model, but did not affect islet blood perfusion (Wu et al. 2012a).

It should be noted that the blood flow lowering effects of GIP were not restricted to the pancreas, as similar effects were observed in other organs supplied by the superior mesenteric and celiac arteries (i.e., the duodenum and liver), both of which supply the pancreas in rats, as well as in colon (supplied by the inferior mesenteric artery), and the adrenals and kidneys. This is in line with previous findings on effects of GLP‐1 on systemic blood flow in normal (Cabou et al. 2008) and transgenic mice (Cabou et al. 2008, 2011), whereas no similar studies have been performed on the effects of GIP. The mechanism for the GIP‐induced vasoconstriction in pancreas and the splanchnic organs is not known. Most of the GIP‐induced effects were seen when high, supraphysiological doses were used, and this was confirmed by our plasma GIP concentration measurements. Thus, it may be that pharmacological effects are most important for the observed effects. However, in view of the possible beneficial effects of decreasing increased islet blood perfusion during conditions of impaired glucose tolerance or overt T2D it may nevertheless be an important finding.

Pretreatment with the ganglion blocker hexamethonium did not attenuate the responses (except for in the kidney) challenging neuron‐mediated effects. This is somewhat surprising since it has been suggested that the peripheral vascular effects of GIP is mediated through the central nervous system (Campbell and Drucker 2013). However, it is still unclear whether exogenously added GIP crosses the blood–brain barrier (Campbell and Drucker 2013). The adenosine receptor antagonist theophylline did not prevent the GIP‐induced decrease in blood flow. It has been suggested that GIP may influence endothelial function (Campbell and Drucker 2013) by affecting nitric oxide (NO) and endothelin‐1; substances to which islets are known to be exquisitely sensitive (Svensson et al. 1994; Lai et al. 2007b).

Our findings in rats are somewhat different from those previously seen in dogs (Fara and Salazar 1978; Kogire et al. 1988, 1992). Since GIP differs somewhat in amino acid sequence this is not surprising. However, its role as an increatin hormone seems unequivocal, but its vascular effects may vary. Differences in reactivity between the celiac artery and the superior mesenteric artery in dogs were seen, with a constriction in the latter and no effects on the former as evaluated by an ultrasonic flowmeter (Kogire et al. 1988). Rather surprisingly, total pancreatic blood flow was decreased up to 17% by GIP, a change of same magnitude as seen in rats in the present study. Both in the canine studies and in our previous studies GIP was administered as a bolus. Subsequent studies in conscious dogs, showed that a *μ*g‐bolus of GIP increased portal blood flow, but decreased blood flow in the hepatic artery (Kogire et al. 1992), which is a branch of the celiac artery. It is well established that GIP receptors are present in blood vessels, preferentially in the endothelium rather than in vascular smooth muscle cells (Usdin et al. 1993; Zhong et al. 2000). In the quoted canine studies (Patzak et al. 2001, 2004; Ruttimann et al. 2009) endothelial cells were isolated from the hepatic artery and portal vein and exposed to GIP. Notably, GIP induced secretion of the vasoconstrictor endothelin‐1, from hepatic artery endothelium with an EC_50_ of 0.3 nmol/L, but had no such effect on portal endothelium, where it instead stimulated formation of nitric oxide (Ding et al. 2004). It should be noted that islet blood flow is regulated at the arteriolar level, not at venous vessels (Brunicardi et al. 1996). Thus, it may be speculated that ET‐1 release occurred also in the rodents studied in the present study.

To further elucidate this we studied the vascular reactivity in arterioles of isolated islets, that is, islets devoid of innervation but containing an intact endothelium (Lai et al. 2007a). We found that high glucose concentrations (16.7 mmol/L) caused a 6–7% increase in arteriolar diameter, whereas no dilation was seen when low glucose (2.8 mmol/L) was added to the medium, thereby confirming previous observations (Lai et al. 2007a). When different concentrations of GIP was added to high‐ or low‐glucose‐containing medium, a dose‐dependent (10^−12^–10^−6^ mol/L) arteriolar dilation up to 11–12% was observed. Thus, the findings in this ex vivo perfusion were opposite to that seen in vivo and argue against a local release of ET‐1 in the islet circulation. This would suggest that direct effects of GIP on islet cells, endocrine, and nonendocrine cells favor vasodilation, whereas in vivo these effects are overridden by systemic effects favoring vasoconstriction. Thus, GIP in vivo mediates vascular contraction, which does not seem to be mediated by the nervous system, adenosine, or constrictor local endothelial mediators. Interestingly two of the three doses of GIP used (10 and 60 ng/kg*min) decreased renal blood flow GLP‐1 infusion induces a rapid natriuretic and diuretic response in humans, through largely unknown mechanisms (Skov 2014; Moroi and Kubota 2015). This response has been mainly seen in overhydration (Moroi and Kubota 2015). Recently, it has been appreciated that GLP‐1 agonists and DPP‐4 inhibitors protect vascular renal endothelium by ameliorating oxidative stress and local inflammatory responses (Ussher and Drucker 2012). In saline‐infused control rats, hexamethonium caused a pronounced increase in renal blood flow by removing the sympathetic tone. In animals given the highest dose of GIP (60 ng/kg*min), hexamethonium administration led to an augmented renal blood flow, suggesting that the flow decrease was indeed partially due to activation of presumably sympathetic nerves.

Some comments on the different vascular effects of intraportal and systemic GIP infusion are warranted. GLP‐1 is known to exert direct effects on portal receptors, and it has been suggested that GIP inhibits glucose production in the liver (Hartmann et al. 1986; Drucker 2013). One drawback with direct intraportal infusions is that the opening of the abdominal cavity leads to decreased blood flow in most of the organs studied (Jansson et al. 2001), which was the case also in the present study. The effect of intraportal infusion of GIP on organ blood flow was minor, and no effect on plasma glucose or insulin concentrations was seen in normoglycemic rats after intraportal GIP infusions. As previously shown systemic plasma insulin concentrations were markedly higher after intraportal than after systemic infusion of glucose (*P* < 0.01). However, this response was decreased by GIP.

Vascular effects of GIP could also theoretically be involved in the rapid improvement in glucose homeostasis after RYGB surgery. The aim of the present study was to investigate the possibility that changes in GIP concentrations after RYGB, may directly affect islet blood flow and thereby contribute to the improved glucose homeostasis. An increased insulin release is usually associated with an acute increase in islet blood flow which prevails until blood glucose is normalized (Jansson et al. 2010). However, during prolonged demands for insulin secretion, such as those seen during impaired glucose tolerance and T2D it is theoretically possible that the sustained blood flow increase adversely affects islet endothelial function (Mattsson 2005; Jansson et al. 2010). A decrease in islet blood flow induced by GIP may therefore have positive effects on islet endocrine function. One should also keep in mind that the hormone concentrations which an organ is exposed to represent the sum of the secreted amount of hormone times blood flow. Therefore, further studies are needed to explore the role of hormonal regulation of blood flow on glucose metabolism.

## Conflict of Interest

There is no conflict of interest for any of the authors.
